# Supplementary material for: Development of Procymidone and Difenoconazole Resistance in Alternaria alternata, the Causal Agent of Kiwifruit Brown Spot Disease
Source: Plants (Basel). 2025 Jul 21;14(14):2245. doi: 10.3390/plants14142245 (PMC12300078; doi:10.3390/plants14142245)
Supplement: Supplementary file 1 [file plants-14-02245-s001.zip › plants-3761698-supplementary.pdf]

Table S1. Cross-resistance between procymidone and difenoconazole with other six fungicides

Isolate	Phenotype	procymidone	iprodione	difenoconazole	tebuconazole	prochloraz	pydiflumetofen	pyraclostrobin	thiophanate-methy
AK-01	Pro^S Dif^S	0.32	0.36	0.76	1.23	2.21	0.37	2.35	>100
AK-08	Pro^S Dif^S	0.43	0.54	0.65	0.92	1.78	0.21	4.31	>100
AK-13	Pro^S Dif^S	0.51	0.43	0.53	1.15	5.69	0.36	13.2	>100
AK-26	Pro^S Dif^S	0.32	0.62	0.32	11.6	32.5	0.42	0.98	>100
AK-210	Pro^S Dif^S	0.26	0.38	0.41	0.85	3.63	0.53	3.54	>100
AK-39	Pro^S Dif^S	0.75	0.98	0.29	1.02	3.91	0.24	21.5	>100
AK-101	Pro^S Dif^S	0.29	0.51	0.26	1.26	0.99	0.57	5.34	>100
AK-116	Pro^S Dif^S	0.31	0.47	0.59	1.35	6.51	0.21	2.15	>100
AK-17	Pro^R Dif^S	16.5	21.3	0.35	1.17	2.54	0.19	3.18	>100
AK-016	Pro^R Dif^S	21.3	18.6	0.37	1.35	1.65	0.58	2.46	>100
AK-22	Pro^R Dif^S	32.1	28.6	0.41	21.1	1.97	0.36	5.78	>100
AK-123	Pro^R Dif^S	26.5	25.4	0.22	0.75	6.42	0.21	1.92	>100
AK-043	Pro^R Dif^S	32.9	29.5	0.38	0.68	4.78	0.37	3.57	>100
AK-036	Pro^S Dif^R	0.23	0.32	11.3	1.25	1.57	0.62	1.28	>100
AK-129	Pro^S Dif^R	0.39	0.37	12.5	2.12	3.24	0.37	13.87	>100
AK-205	Pro^S Dif^R	0.39	0.45	20.1	0.95	2.61	0.16	24.12	>100
AK-024	Pro^S Dif^R	0.48	0.55	15.8	1.46	25.4	0.47	4.57	>100
AK-139	Pro^S Dif^R	0.65	0.77	17.2	1.02	2.38	0.39	2.13	>100
AK-163	Pro^R Dif^R	21.38	30.12	21.2	0.98	6.51	0.51	6.21	>100
AK-037	Pro^R Dif^R	29.57	32.62	13.4	2.31	3.22	0.32	1.98	>100

Table S2 Characteristics of fitness for procymidone- and difenoconazole -sensitive and -resistant isolates

Isolate	Phenotype	Growth (mm/d)	Sporulation ($\times10^4$ conidia/mL)	Germination (%)	Pathogenicity (Lesion diameter/cm)	CFI
AK-01	Pro^S Dif^S	6.2 b	5.1c	100.0 a	4.4 b	13912.8c
AK-08		5.4 c	7.3a	97.3 b	4.3 b	16492.9b
AK-13		4.9 c	4.6c	97.1 b	4.9 a	10724.3c
AK-26		7.3a	7.5a	97.2b	3.6c	19518.1a
AK-210		5.5c	6.3b	99.4a	5.1a	17565.5b
AK-17	Pro^R Dif^S	4.4	5.2 c	97.3 b	5.1a	11353.7c
AK-016		5.1 c	7.7a	99.4 a	4.2b	16394.4b
AK-22		6.2 b	4.6 c	100.0a	3.3c	9411.6c
AK-123		6.1b	6.1b	97.4b	5.4a	19570.9a
AK-043		4.8c	5.3c	95.3c	4.3b	10425.1c
AK-036	Pro^S Dif^R	6.1 b	3.9 d	99.3 a	5.2a	12284.2c
AK-129		5.3 c	4.8 c	99.7 a	3.6c	9130.9c
AK-205		5.2 c	7.5a	100.0 a	3.5 c	13650.0c
AK-024		7.1a	5.1c	97.2b	2.5d	8799.0c
AK-139		5.2c	6.1b	100.0a	5.3a	16811.6b
AK-163	Pro^R Dif^R	5.3 c	4.1d	95.2c	2.2d	4551.1d
AK-037		5.2 c	3.9d	91.4d	2.2d	4077.9d

*Pro^S, Pro^R, Dif^S, and Dif^R mean procymidone-sensitive, procymidone- resistant, difenoconazole -sensitive, and resistant to difenoconazole, respectively. CFI = Mycelial growth × Conidia production × Conidial germination × Lesion diameter. **Mean values in the same column with the same letters were not statistically different ($P > 0.05$) according to the least significance difference (LSD) test.

Table S3 Primers and sequences

**Primers**	**Sequences (5′-3′)**	**Reference**
OS1-1(F)	TGTGGCTTGCGGTTATT	Wang et al. 2021
OS1-1(R)	GACTTGACTGGCAAACTCC	
OS1-2(F)	GGACATCGGCGCACTGAAAAGGG	
OS1-2(R)	GTGACAAAATCTCACCCTGAAC	
OS1-3(F)	GCAGTTTGCTCACGA AGTCACC	
OS1-3(R)	ACCCATGAGAGTGACGAGACG	
OS1-4(F)	GCCGTTTCCGACACCGGAATAGG	
OS1-4(R)	GGCTTCTAATCAAGCCTTCTCCTC	
AaCYP51-for	GCATGCCGGTCCCTGCCAAG	Feng et al. 2025
AaCYP51-rev	CGGCATTCAACTCGAATAAC	

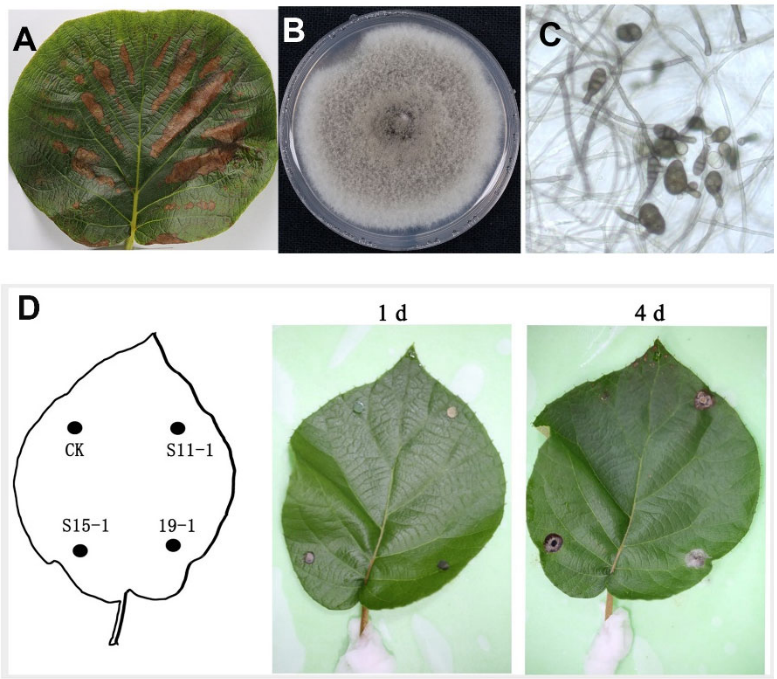

Figure S1. Pathogenicity and morphological characteristics of *Alternaria alternata* causing kiwifruit brown spot disease

A, Symptoms of brown spot disease in the field. B, Mycelium colony on PDA plate. C, conidium morphology. D, Pathogenicity of isolates S11-1,S15-1 and 19-1.
